# Perisomatic innervation and neurochemical features of giant pyramidal neurons in both hemispheres of the human primary motor cortex

**DOI:** 10.1007/s00429-020-02182-8

**Published:** 2020-12-23

**Authors:** Péter Szocsics, Péter Papp, László Havas, Masahiko Watanabe, Zsófia Maglóczky

**Affiliations:** 1 Human Brain Research Laboratory, Institute of Experimental Medicine, ELKH, Budapest, Hungary; 2 Szentágothai János Doctoral School of Neuroscience, Semmelweis University, Budapest, Hungary; 3 Laboratory of Cerebral Cortex, Institute of Experimental Medicine, ELKH, Budapest, Hungary; 4 Department of Pathology, Szt. Borbála Hospital, Tatabánya, Hungary; 5 Department of Anatomy and Embryology, Hokkaido University, Sapporo, Japan

**Keywords:** Human, Post-mortem, Primary motor cortex, Betz cells, Parvalbumin, Neurochemical features, Innervation

## Abstract

Betz cells—the gigantopyramidal neurons found in high amount in the primary motor cortex—are among of the most characteristic neuronal cells. A part of them contains the calcium-binding protein parvalbumin (PV) in primates. However, less is known about these cells in the human motor cortex despite their important role in different neurological disorders. Therefore, the aim of our study was to investigate the neurochemical features and perisomatic input properties of Betz cells in control human samples with short post-mortem interval. We used different microscopic techniques to investigate the primary motor cortex of both hemispheres. The soma size and density, and expression of PV of the Betz cells were investigated. Furthermore, we used confocal fluorescent and electron microscopy to examine their perisomatic input. The soma size and density showed moderate variability among samples and hemispheres. Post-mortem interval and hemispherical localization did not influence these features. Around 70% of Betz cells expressed PV, but in less intensity than the cortical interneurons. Betz neurons receive dense perisomatic input, which are mostly VIAAT- (vesicular inhibitory amino acid transporter) and PV immunopositive. In the electron microscope, we found PV-immunolabelled terminals with asymmetric-like synaptic structure, too. Terminals with morphologically similar synaptic specialisation were also found among vGluT2- (vesicular glutamate transporter type 2) immunostained terminals contacting Betz cells. Our data suggest that Betz cells’ morphological properties showed less variability among subjects and hemispheres than the density of them. Their neurochemical and perisomatic input characteristics support their role in execution of fast and precise movements.

## Introduction

Motor control is crucial for reaction and survival in the environment, and the primary motor cortex (Brodmann’s area 4—BA 4) plays a major role in it. However, its functional contribution in movement control is not yet fully understood (Omrani et al. 2017). One study in pig-tailed macaques showed that the primary motor cortex produces around 50% of the corticospinal projections originating from the frontal lobe (Dum and Strick 1991). This region differs from other areas of the neocortex in some peculiar features. According to Brodmann, it is found in the caudal part of the precentral gyrus in the human brain (Brodmann 1909). This region Brodmann’s area 4 (BA 4) receives input from several motor and sensory areas (e.g. postcentral sulcus or superior parietal lobule), and subcortical structures as well (e.g. ventrolateral (VL) nucleus of the thalamus, as a significant parvalbumin-containing input) (Dum and Strick 2002; Fang et al. 2006; Gandolla et al. 2014; Paxinos and Mai 2003).

Since the study of Penfield and Boldrey, it is known that motor control follows a somatotopical organisation in BA 4 (Penfield and Boldrey 1937). Electrical stimulation of the area causes stereotyped movements on the periphery like grimacing, vocalisation or cooperative movements of the digits (Catani 2017). BA 4 shows some special characteristics in cytoarchitecture as well. First, the inner granular cortical layer (layer 4) is thin but exists in this region (Barbas and García-Cabezas 2015; Bopp et al. 2017). Furthermore, this region has a special population of cells: the giant pyramidal neurons, also called Betz cells (Brodmann 1909).

The giant pyramidal cells of the primary motor cortex have garnered wide scientific interest ever since their original discovery in 1874 by Vladimir Betz (Betz 1874). Due to their specific location and conspicuous size, these neurons can be easily distinguished from other cell populations. Therefore, these giant pyramidal neurons, or Betz cells, have been actively studied in several species, to explore their morphology, structure, electrophysiological properties and synaptic connections (Jacobs et al. 2018; Kaiserman-Abramof and Peters 1972; Rivara et al. 2003; Sasaki and Iwata 1999, 2001; Tigges et al. 1992). However, this cell population is not distinguishable in mice and rats, in the two most widely used model animals in laboratory practice (Jacobs et al. 2018; Oswald et al. 2013). Therefore, their proper scientific investigation is complicated, despite their crucial role in neurological diseases, such as amyotrophic lateral sclerosis (Sasaki and Iwata 1999) and multiple system atrophy (Colosimo et al. 2005).

Beside their large size—60–110 µm in length (Braak and Braak 1976)—,these cells can be differentiated from other pyramidal cells by various additional factors, such as having a high-contrast nucleolus, a large amount of rough endoplasmic reticulum and lipofuscin particles, possessing circumferential dendrites which may originate from any parts of the soma (Rivara et al. 2003). Furthermore, Betz cells possessing proximal bifurcating apical dendrites are described in numerous species, and these neurons show a huge individual diversity in cellular and dendritic morphology (Jacobs et al. 2018).

According to electron microscopic analysis of the human pyramidal tract (Graf von Keyserlingk and Schramm 1984) ~ 1.4% of the corticospinal fibres are large in diameters (more than 10 µm). We can assume that the majority of them are originating from the giant pyramidal neurons, including the fibres from BA 4. Roles of the Betz cells are not fully understood (Jacobs et al. 2018). Due to localization and electrophysiological properties, these neurons most likely contribute to phasic control of antigravity muscles (Scheibel et al. 1974) and other fine and precise movements (Venkadesan and Valero-Cuevas 2008; Perge et al. 2012). Studies carried out on rhesus macaques showed that the axons of larger size pyramidal tract neurons are capable of higher conduction velocity (Vigneswaran et al. 2011). As for the neurochemical background of this phenomenon, a proportion of the giant pyramidal cells are presumed to contain a type of calcium-binding protein, parvalbumin (PV) in monkeys (Preuss and Kaas 1996), and the Kv3.1 potassium channel (Ichinohe et al. 2004). The PV labelling in these pyramidal cells was significantly lower compared to interneurons (Preuss and Kaas 1996). Parvalbumin operates via its special calcium buffer properties (Schwaller 2010), while Kv3.1 is a rapidly functioning potassium channel (Kaczmarek and Zhang 2017). Both have a functional role in fast-spiking characteristics, as described in studies with primates (Preuss and Kaas 1996; Ichinohe et al. 2004) and mice (Akemann et al. 2004). Calcium-binding proteins, e.g. parvalbumin, calbindin and calretinin are present in distinct groups of interneurons and some pyramidal cells in the neocortex across several species (Hof et al. 1999). In the case of calbindin, this protein was found in a subgroup of layer 3 pyramidal cells in the human neocortex (Hayes and Lewis 1992) and in granule cells and principal cells of human hippocampus (Seress et al. 1993). Calretinin containing pyramidal cells were described in several hominids, including humans, in the anterior cingulate cortex (Hof et al. 2001).

Betz cells receive a vast amount of perisomatic input ~ 260 terminals/cell in rhesus monkey (Tigges et al. 1992) and 870/cell in cats (Kaiserman-Abramof and Peters 1972)—far more than was observed on other pyramidal cells’ surface of the same area (Sloper et al. 1978). Most of these terminals originate from the large PV-containing basket neurons—GABAergic interneurons—of the same region. Several authors assumed these basket cells are the only sources of the perisomatic terminals (Tigges et al. 1992; Sloper et al. 1978). However, Marin-Padilla suggested that axons with thalamic origins might end on the soma of Betz cells, at least in case of human infants (Marin-Padilla 1972). In addition, the neurons of the ventrolateral (VL) thalamus—projecting to the primary motor cortex—contain parvalbumin (Munkle et al. 2000).

In summary, a significant number of studies have profoundly explored the neurochemical and synaptic features of giant pyramidal cells in various non-human species; however, no sufficient evidence has been accumulated so far about human samples. Therefore, the aim of our study has been to investigate the neurochemical characteristics of giant pyramidal cells in good quality, properly fixed human brain samples with short post-mortem interval. Our special interest is to determine whether human Betz cells also express PV, and if they do, in what proportions. Another goal of our study carried out using double-immunofluorescent and electron microscopic examinations is to confirm whether perisomatic terminals originating from cell types other than inhibitory interneurons, innervate the soma of Betz cells.

## Materials and methods

### Human samples

Human primary motor cortex samples were obtained from seven subjects who died due to causes not directly involving any brain disease or damage, and without having medical history of neurological or psychiatric disorder either. All procedures were carried out in compliance with the Declaration of Helsinki and approved by the Regional Committee of Science and Research Ethics of Scientific Council of Health (ETT TUKEB 31443/2011/EKU, renewed: ETT TUKEB 15032/2019/EKU). The subjects went through autopsy in the Department of Pathology of St. Borbála Hospital, Tatabánya. Informed consent by relatives was obtained for the use of brain tissue and for access to medical records for research purposes.

Brains were removed after death and the internal carotid and vertebrate arteries were cannulated. First, physiological saline containing 0.33% heparin was perfused through this system (1.5 l in 30 min), after which perfusion continued with Zamboni fixative solution containing 4% paraformaldehyde, 0.05% or 0% glutaraldehyde and 0.2% picric acid in 0.1 M phosphate buffer (PB, pH = 7.4, 2 h, 4 L). Post-mortem interval (PMI) was determined between the time of death and the start of perfusion by Zamboni fixative. Table 1 shows the important parameters of subjects and perfusions.

**Table 1:** General data of the subjects

Subject	SKO3	SKO9	SKO11	SKO13	SKO17	SKO19	SKO20	Average
Gender	Female	Female	Male	Female	Female	Female	Male	
Age	59 years	78 years	77 years	60 years	75 years	61 years	27 years	64 ± 17 years
Post-mortem interval	5 h 5'	3 h 45′	2 h 55′	3 h 25′	4 h 35′	2 h 53′	3 h 35′	3 h 40′ ± 52′
Brain mass	n.a	n.a	1205 g	1250 g	1100 g	1050 g	1540 g	1263 ± 177 g

**Fig. 1:**
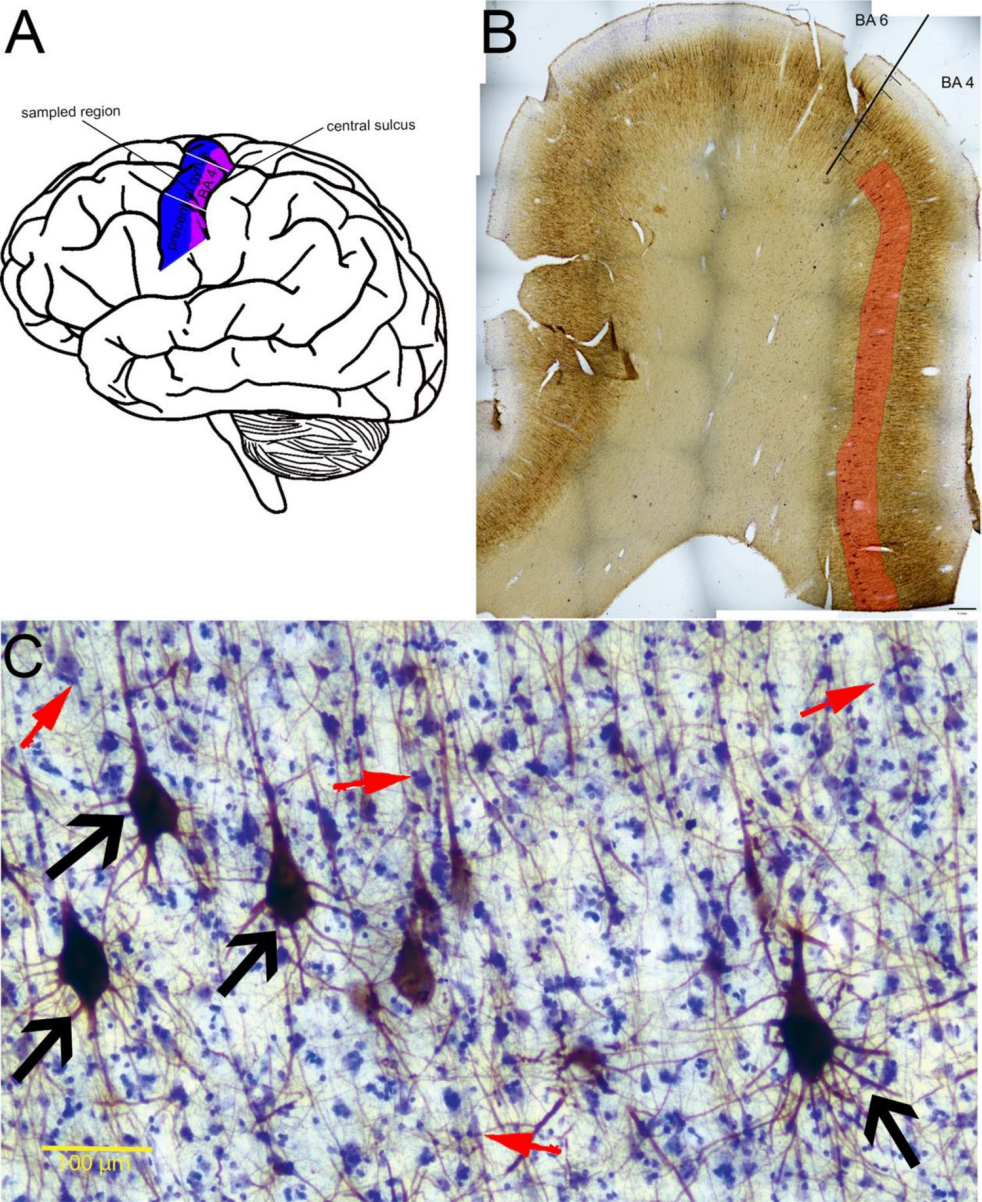
**a** Schematic representation of the sampling site of the primary motor area examined in the present study in the brain. Precentral gyrus is labelled with blue, BA 4 with purple. Our samples were originating between 15 and 45 mm from the medial line (between the white lines in the picture. Source of the drawing: https://freesvg.org/vector-image-of-side-view-of-human-brain-in-pink, under Public Domain. **b** low magnification photomicrograph of the precentral gyrus immunolabelled with SMI32 and counterstained with Cresyl violet. The border between BA 4 and 6 is indicated. The red highlighting shows the sampling area inside layer 5 of the BA 4. Scale: 1 mm. **c** SMI32-immunolabeled section of layer 5 in the human primary motor cortex counterstained with Cresyl violet. The giant pyramidal cells or Betz cells (some indicated by black arrows) are labelled by both methods, while a proportion of the other pyramidal cells are only visible with Cresyl violet (red arrows). Scale: 100 µm

Tissue blocks were cut out from the precentral gyrus of both hemisphere 15–45 mm lateral from the interhemispheric fissure (Fig. 1). The caudal part of the gyrus contains the primary motor cortical region [BA 4 (Brodmann 1909)]. The borders of the BA 4 region are detailed below. The sampling site is in the upper limb representing region (Penfield and Boldrey 1937) with small overlap of the lower limb and face areas, respectively (Catani 2017). A previous study showed the heterogeneity of giant pyramidal neurons along the precentral gyrus (Rivara et al. 2003); therefore, it was important to have samples from the same subregion in each subject. This part contains the Betz cells in the highest density (Rivara et al. 2003). The blocks were post-fixed overnight in the same fixative without glutaraldehyde at 4 °C.

### Immunohistochemistry

For immunohistochemistry, we used similar methodology to what was previously reported in detail by our research group (Tóth et al. 2010). After sampling, 60-µm-thick vibratome sections were cut from the blocks and prepared for immunohistochemistry. Sections were washed four times in 0.1 M PB before being immersed in 30% of sucrose in 0.1 M PB for 1–2 days. Sections were then freeze-thawed three times over liquid nitrogen and washed in 0.1 M PB again. Endogenous peroxidase activity was blocked using 1% H_2_O_2_ in TRIS-buffered saline (TBS) for 10 min. TBS was used for each washing (three times for 10 min), between sera, and for dilution of the antibodies. Non-specific immunoglobulin binding was blocked using 4% bovine serum albumin (BSA) in TBS for 45 min. The sections for fluorescent imaging were additionally treated with 0.1% Triton, to increase membrane permeability. For visualisation, we used 3,3′-diaminobenzidine-tetrahydrochloride (DAB) chromogen and DAB intensified with ammonium nickel sulphate (DAB-Ni) or immunofluorescent techniques detailed later. In each experiment, we prepared 2–4 non-consecutive sections from a sample by the rules of systematic random sampling. Fourteen samples from both hemispheres of the 7 subjects were incubated for DAB reaction with anti-SMI32 primary antibody (Biolegend, mouse, 1:4000) for two consecutive nights during constant agitation at 4 °C, to determine the giant pyramidal cells’ density. Afterwards, the samples were incubated in biotinylated anti-mouse secondary antibody (Vector, 1:250, 2 h), then in avidin-biotinylated horseradish peroxidase complex (ABC, Vector, 1:250, 1.5 h). The next step was the preincubation with DAB for 20 min, followed by development using 0.01% H_2_O_2_. Before dehydration, sections were incubated in 0.5% OsO_4_ in PB for 10 min. Dehydration comprised a 50% to absolute ethanol series, with an additional step of uranyl acetate at the 70% ethanol stage for 30 min. Sections were mounted in Durcupan (ACM, Fluka).

Control samples from the primary motor cortex were prepared with similar methodology but with anti-parvalbumin primary antibody (Swant, mouse, 1:5000) for electron microscopical analysis to visualise parvalbumin-containing perisomatic input.

An earlier study (Tsang et al. 2000) showed that non-phosphorylated neurofilament SMI32 is present in Betz cells, and we checked whether we found the same expression profile in the samples with our fixation method. Therefore, two primary motor cortical samples were mounted in chrome-gelatine after development, and counterstained with Cresyl violet to investigate whether all of the giant pyramidal neurons were SMI32-immunopositive.

We used the more specific DAB-Ni protocol to visualise terminals of the subcortical glutamate input. Two samples were incubated with anti-vesicular glutamate transporter type 2 (vGluT2) antibody (mouse, Chemicon, 1:1000) for two nights at 4 °C to show the subcortical glutamate-containing afferents. Biotin-SP (long spacer, donkey anti-mouse, 1:600, Jackson) secondary antibody was applied for 2 h, then sections were incubated with ABC (Vector, 1:500) for 1.5 h. DAB-Ni was used as chromogen, with 20-min preincubation. The dehydration and mounting process was the same as the DAB method detailed above.

Fluorescent immunohistochemistry was used to measure the size of Betz cells and for co-expression studies. After blocking, the following primary antibodies were used: anti-NeuN (Chemicon, mouse, 1:2000) or anti-SMI32 (Biolegend, mouse, 1:4000) with anti-PV (Swant, rabbit, 1:5000), anti-PV (Swant, mouse, 1:5000) with anti-VIAAT [vesicular inhibitory amino acid transporter, goat, gift of Masahiko Watanabe, 1:2000 (Iwakura et al. 2012; Kudo et al. 2012)] or anti-vGluT1 (vesicular glutamate transporter type 1, Synaptic Systems, rabbit, 1:10,000). Sample sizes are listed in Table 2. After 2 days of incubation, the following fluorescent secondary antibodies were applied: Alexa 488 donkey anti-mouse [Molecular Probes, 1:500—for labelling NeuN (mouse), SMI32 (mouse), PV (mouse)]. Alexa 594 donkey anti-rabbit [Molecular Probes, 1:500—for labelling PV (rabbit), vGluT1 (rabbit)] and Alexa 594 donkey anti-goat [Molecular Probes, 1:500—for labelling VIAAT (goat)]. The samples were incubated in darkness for 3 h. All primary and secondary antibodies were tested by the producers for specificity. Next, tissue autofluorescence was reduced (Schnell et al. 1999) with acidic (pH = 5) 3 mmol/l cupric sulphate and 50 mmol/l ammonium acetate dilution in distilled water. Incubation was carried out in the dark for 40 min. Before and after the incubation, samples were rinsed briefly in distilled water. Finally, sections were mounted in TBS, covered up by antifade medium Vectashield (Vector) and stored at 4 °C.

**Table 2:** Sample sizes for different experiments

Experiment	Labelling	Samples	
Measurement of density using anti-SMI32 immunostaining	DAB	3–4 sections per both hemispheres per 7 subjects	All subjects
Cell size measurement using anti-NeuN	Immunofluorescent	2–3 sections per both hemispheres per 7 subjects	All subjects
Measurement of PV-expression in Betz cells	Immunofluorescent	2–3 sections per both hemispheres per 4 subjects	SKO3, SKO9, SKO11, SKO20
Measurement of VIAAT and vGlut1 co-expression with PV	Immunofluorescent	2 sections from left hemispheres per 2 subjects	SKO11, SKO19
Measurement of vGlut2-immunopositive terminals	DAB-Ni	2 sections from different hemispheres per 2 subjects	SKO11 (left), SKO20 (right)

*DAB *3,3′-diaminobenzidine-tetrahydrochloride chromogen, *DAB-Ni* DAB chromogen with ammonium nickel sulphate, *NeuN* neuronal nuclei, *PV* parvalbumin, *VIAAT* vesicular inhibitory amino acid transporter, *VGluT1 and -2* vesicular glutamate transporter type 1 and 2, *SKO#* control subject ID

### Electron microscopy

PV-DAB- and vGluT2-DAB-Ni-immunolabelled sections were prepared for electron microscopic analysis. Small ROIs (region of interest) were cut out around identified Betz cells (1–3 cells) from layer 5. Sixty nanometers ultrathin slices were prepared and put on single slot copper grids. The samples were investigated using a transmission electron microscope; we were searching for perisomatic terminal profiles labelled by the chromogens.

### Collecting data and statistical analyses

#### Density measurement in both hemispheres

BA 4 region was determined by its histological properties, differentiating it from the neighbouring regions (caudal: BA 3a and rostral: BA 6). The subregions inside the primary motor cortex [BA 4a and 4p (Geyer et al. 1996)] were not distinguished from each other. BA 3a is a highly granular region, with thick layer 4 (Brodmann 1909). Our sections did not contain BA 3a, thus, the sampling area started from the edge of the sections. The border of BA 4 and 6 was determined by the lack of Betz cell clusters and the transition of the layer 3’s pyramidal cells to a larger morphology (Rivara et al. 2003). The sampling area did not reach this cytoarchitectonical border of BA 4 and 6 (Fig. 1).

BA 4 layer 5 of the SMI32-immunolabelled sections were digitalized using Nikon Ci-L microscope in light mode with 10 × objective (Plan Achromat, NA = 0.25), DS-Fi3 microscope camera and NIS Elements BR acquisition software. Distinct images from the same section were merged with Photoshop CS6 graphics software (Adobe) and analysed in Fiji Image J software. The borders between layers were identified with immunohistochemical and cellular characteristics. Layer 4 is very thin in this region and does not contain SMI32-immunopositive cells (Barbas and García-Cabezas 2015; Bopp et al. 2017). Therefore, layer 3–5 border was defined by the hiatus between labelled pyramidal cells. The layer 5–6 border was identified by the neuronal cell morphology shift from “usual” pyramidal to a more diverse morphology (Meyer 1987) and the absence of Betz cells. Lateral edges were on the edge of the slice or before the transition to BA 6 (Fig. 1). After the identification of layer 5, Betz cell bodies were counted on the images. Betz cells were identified by the following characteristics: large soma size, dendritic branch circumstantial from the whole cell body, large amount of lipofuscin, and localization in 5b sublayer (Rivara et al. 2003). Cell density was determined by the number of counted cells divided by the layer area (N/mm^2^). Mean densities were determined as follows: the total cell numbers were divided by the investigated area in each sample.

##### Soma size

NeuN-immunolabelled fluorescent sections were microphotographed with Nikon Ci-L microscope in wide-field fluorescent mode with 40 × objective (Plan Fluor, NA = 0.75), DS-Fi3 camera and NIS Elements BR acquisition software. The whole section was examined and the area (µm^2^) of every Betz cell body with visible nucleus was measured at its largest extension.

##### Determination of the PV content of Betz cells

SMI32 (mouse, visualised by Alexa 488 secondary antibody) and PV (rabbit, visualised by Alexa 594 secondary antibody) primary antibodies were used for double-immunofluorescent labelling. For the acquisition, we used the same settings as detailed above. Betz cell bodies were identified based on SMI32 immunofluorescence. Microphotographs of the SMI32 and PV channels were merged and analysed with Fiji Image J software. We measured the soma area and investigated SMI32-PV co-expression in Betz neurons.

##### Co-expression of PV- and VIAAT or vGluT1

Betz cell bodies of two samples (SKO11, SKO19, left hemisphere) were microphotographed with a Nikon C2 confocal microscope, with 20 × objective (Plan Apo VC NA = 0.75) and NIS Elements AR acquisition software (version: 4.30) using 488-nm and 561-nm laser lines. Giant pyramidal cells were determined according to the characteristics listed above. Cell bodies were identified by their PV immunopositivity. We counted the terminals contacting the cells’ somata, measured their area and determined their PV, VIAAT, or vGluT1 content. A contact was confirmed when no hiatus could be observed between the cell body and terminals in the same focal plane.

### Statistical analyses

All data were collected in Microsoft Excel, and Statistica 13.4 (TIBCO Software) was used for statistical analyses. For comparison between samples, we used non-parametric Mann–Whitney *U* tests. Betz cells’ density and soma size data were tested for putative associations with post-mortem interval (PMI) of the perfusion using multiple regression analyses. Furthermore, we checked whether lateralization has an influence on these features using Mann–Whitney’s *U* tests. Table 3 shows the number of cells and terminals in each comparison.

**Table 3:** Number of measured cells and terminals by subject and hemisphere in different experiments

Subject	SKO3	SKO9	SKO11	SKO13	SKO17	SKO19	SKO20	
Experiment	Number of measured elements							SUM
NeuN + Betz cells (fluorescent, left/right)	154/83	35/26	31/32	27/29	27/32	38/80	20/31	332/313
SMI32 + Betz cells (DAB, left/right)	55/69	56/43	104/109	64/185	35/36	45/60	83/56	442/558
Proportion of PV + Betz cells (fluorescent, left/right)		38/23	46/62	54/34			58/37	196/156
VIAAT-PV co-localisation in perisomatic terminals (fluorescent, terminals/cells)			214/23			101/26		315/49
vGluT1-PV co-localisation in perisomatic terminals (fluorescent, terminals/cells)			185/24			125/27		310/51

*DAB *3,3′-diaminobenzidine-tetrahydrochloride chromogen, *NeuN* neuronal nuclei, *PV* parvalbumin, *VIAAT* vesicular inhibitory amino acid transporter, *VGluT1 and -2* vesicular glutamate transporter type 1 and 2, *SKO#* control subject ID

## Results

We have examined the morphological features and input characteristics of the Betz cells in the area of the human primary motor cortex, approximately in the somatotopic upper limb area of the motor homunculus (Penfield and Boldrey 1937). The size of the cell body, density of cells, and parvalbumin content were compared in both sexes in the left and right hemispheres. Cortical and subcortical inputs to Betz cells were analysed by vesicular inhibitory amino acid (VIAAT) and glutamate transporters type 1 and 2 immunostainings (VGluT1 and -2 expressions allow differentiation between cortical and subcortical inputs, respectively).

### Size and density of giant pyramidal neurons: hemispherical and intersubject differences

The size of Betz cells was determined with 20 × magnification images, using NeuN immunofluorescent labelling. The giant pyramidal cells were identified based on their size, localization (5b sublayer), higher lipofuscin content and proximal dendritic morphology (Rivara et al. 2003).

It is known that SMI32 antibody-labelling non-phosphorylated neurofilament H is present only in a subset of layer 5 pyramidal cells (Campbell and Morrison 1989). However, one study (Tsang et al. 2000) showed that all Betz cells are expressing SMI32. Therefore, we have checked the samples in two subjects, which were immunostained with SMI32 and counterstained by Cresyl violet (Nissl staining) whether all giant pyramidal neurons are immunolabelled for PV with our fixation method, too. We found that all Betz cells’ bodies were SMI32-immunopositive (Fig. 1). Thus, SMI32 proved to be a reliable marker to determine the density of Betz cells.

**Fig. 2:**
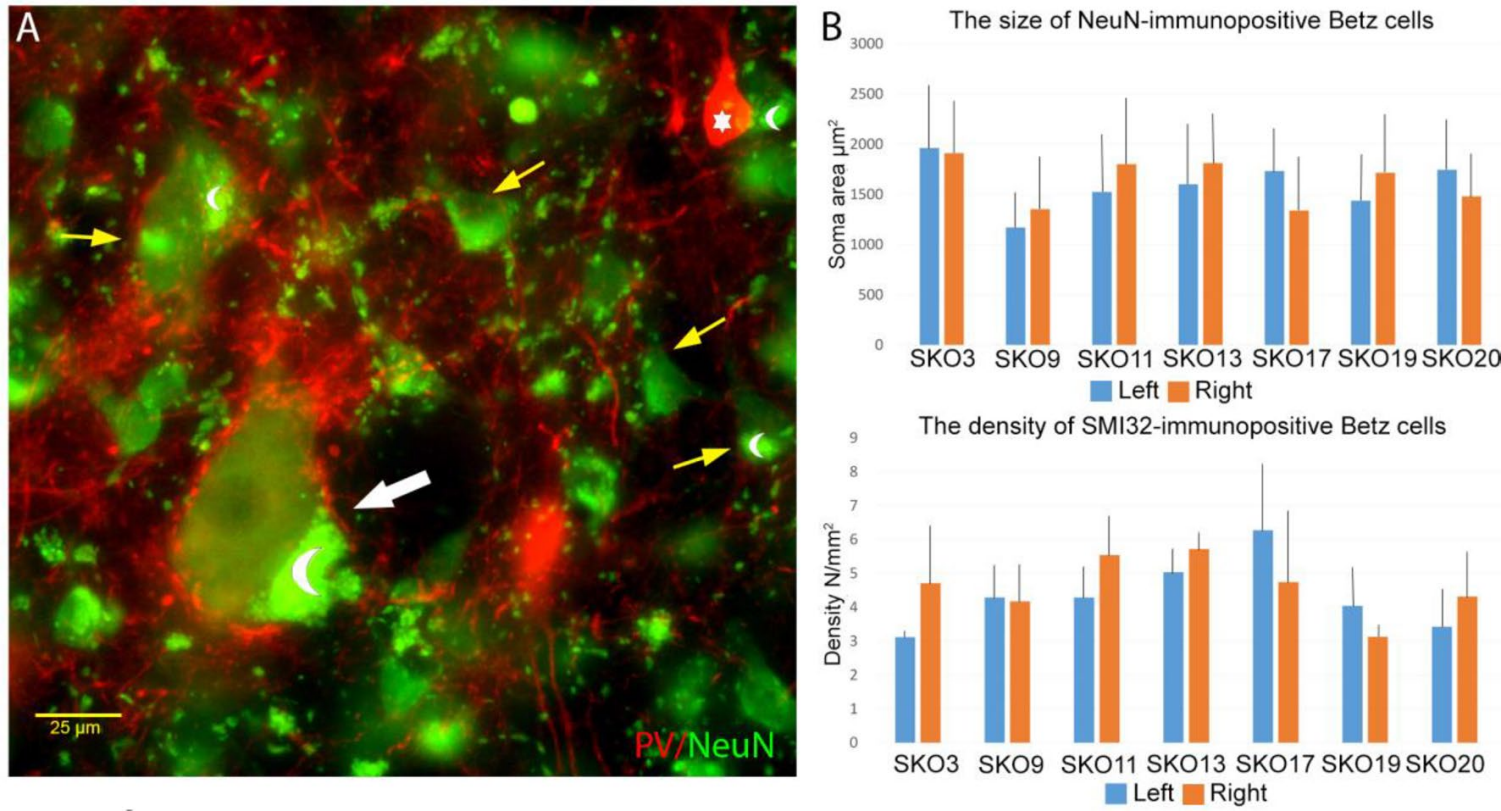
Size and density of Betz cells. **a** Wide-field fluorescent micrograph of layer 5 of the primary motor cortex (BA 4) with NeuN and PV immunolabellings (green and red, respectively). White arrow shows a giant pyramidal cell; note the dense coverage by PV-immunopositive terminals around the faintly PV-labelled soma. Yellow arrows are pointed to other pyramidal cells with smaller size. White star marks a PV-immunopositive interneuron, crescent moon symbols show lipofuscin granules. **b** Diagrams of size and density measurements of the giant pyramidal neurons. The soma area and density of the Betz cells hugely varied among subjects and hemispheres. Multiple regression analyses and Mann–Whitney *U* tests did not show any relationship between the size or density and age, post-mortem interval, hemispheres or gender. Blue columns indicate left and orange columns indicate right hemispheres. Black lines show standard deviation. For details, see Table 4

The mean soma size of NeuN-labelled Betz cells showed moderate variability among subjects and hemispheres (Fig. 2, Mean_left_ = 1596 ± 254 µm^2^, Mean_right_ = 1629 ± 236 µm^2^; mean difference between hemispheres: 237 µm^2^). The smallest mean soma size (SKO9_left_ = 1170.7 µm^2^) was 60% of the largest mean soma size (SKO3_left_ = 1961.6 µm^2^). Multiple regression analysis did not show any relationship between soma size and PMI (Table 4). According to the Mann–Whitney’s *U* test, lateralization did not influence the soma size of Betz cells.

**Table 4:** Correlation probes with the mean size of Betz cells

Measured parameters		Number of cases	b*	Std. Err. of b*	*p* value
Mean size/PMI	All	14	0.294	0.276	0.307
Multiple regression analysis	Left	7	0.620	0.351	0.138
	Right	7	− 0.054	0.447	0.909
Mean density/PMI	All	14	0.025	0.289	0.931
Multiple regression analysis	Left	7	− 0.035	0.447	0.941
	Right	7	0.099	0.445	0.833
Mean size/hemispheres Mann–Whitney’s *U* test		Mann–Whitney’s *U* = 23.00; *N*1 = 7; *N*2 = 7; *p* = 0.902 (two sided)			
Mean density/hemispheres Mann–Whitney’s *U* test		Mann–Whitney’s *U* = 18.00; *N*1 = 7; *N*2 = 7; *p* = 0.456 (two sided)			

Statistical analyses of soma size and density of Betz cells. Multiple regression analyses did not show significant changes by post-mortem interval (PMI). Mann–Whitney analyses did not show hemispherical differences

Cell density was determined as the number of SMI32-positive neurons per unit area (mm^2^). Betz cells were identified by the same criteria described above. The mean density of SMI32 labelled Betz cells showed moderate variance among subjects and hemispheres. (Fig. 2, Mean_left_ = 4.35 ± 1.05/mm^2^, Mean_right_ = 4.62 ± 0.88 1/mm^2^; Mean difference between hemispheres: 1.00/mm^2^). The smallest mean cell density (SKO3_left_ = 3.12/mm^2^) was 50% of the largest one (SKO17_left_ = 6.28/mm^2^). Multiple regression analyses did not show any relationship between the gigantopyramidal cells’ density and PMI (Table 4). According to the Mann–Whitney’s *U* tests, lateralization did not influence the density of Betz cells.

### Parvalbumin expression of Betz cells

**Fig. 3:**
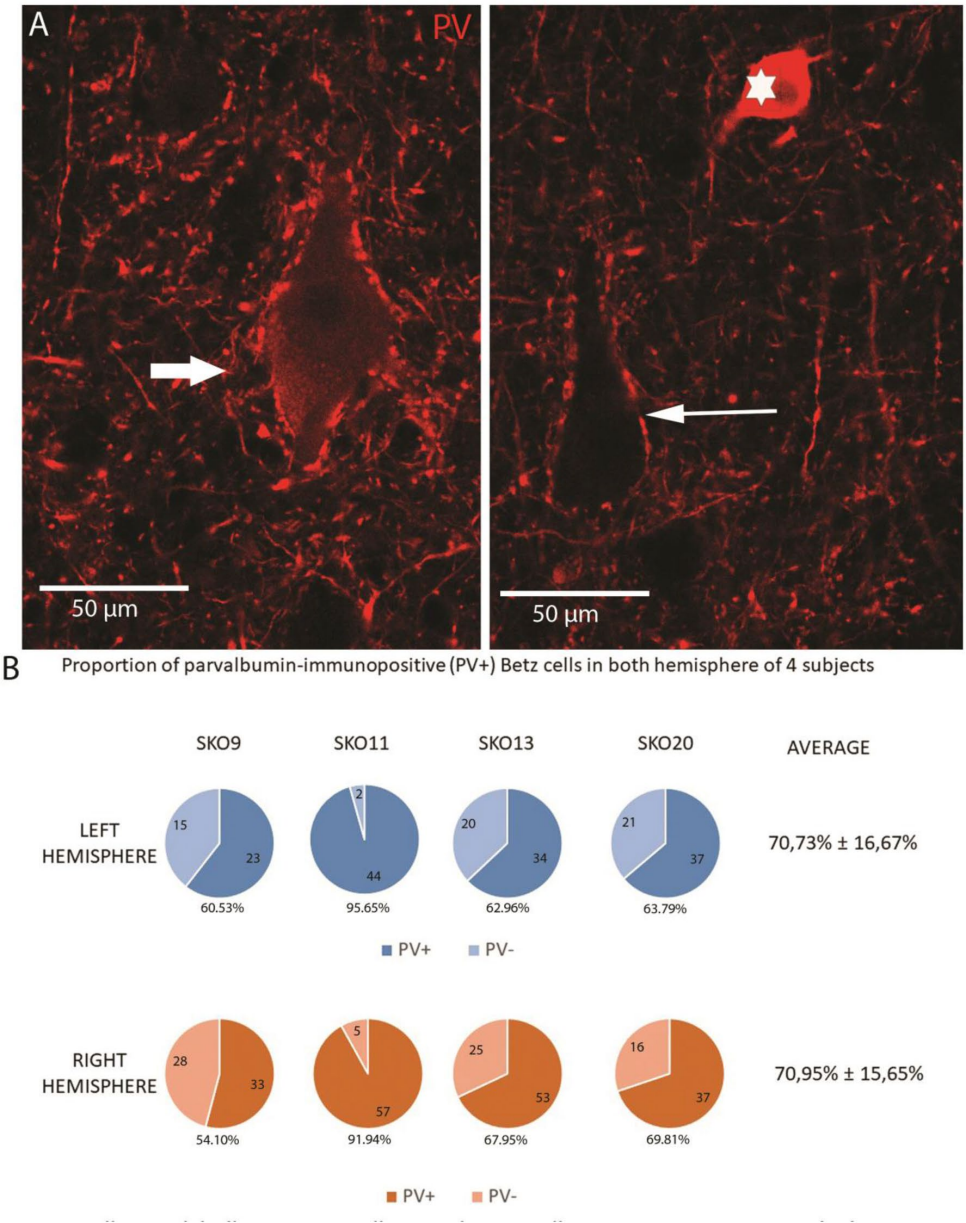
Parvalbumin labelling in Betz cells. **a** Parvalbumin-immunopositive (thick arrow) and parvalbumin-immunonegative (thin arrow) Betz cells on confocal fluorescent images with 20 × magnification. The parvalbumin expression of the Betz cells is lower compared to interneurons (one PV-immunopositive interneuron is indicated with a white star). **b** The proportion of PV-immunopositive and -negative cells in four control subjects in both hemispheres

To check whether the giant pyramidal neurons express PV, we have carried out double-fluorescence immunostaining for SMI32 and PV. Betz cells were identified based on their size, morphology and localization with SMI32. Most of the Betz cells showed parvalbumin-immunopositivity, but the proportion of PV-immunopositive Betz cells varied among samples (Fig. 3). In extreme cases, almost all giant pyramidal neurons expressed parvalbumin (SKO11_left_: 95.65%, SKO11_right_: 91.94%), however, in general approximately 70% of the cells showed PV immunoreactivity. The parvalbumin signal was found to be less intensive in Betz cells compared to interneurons or PV-immunopositive terminals but was clearly distinguishable from the background (Fig. 3). The mean soma size of PV-expressing Betz cells was larger in both hemispheres of all examined subjects (Mean difference: 20.80 ± 16.61%) than the PV-negative Betz cells. During our investigation, we did not find SMI32-immunolabelled pyramidal cells which do not meet the morphological criteria of Betz cells and showed PV immunopositivity.

### Perisomatic terminals

**Fig. 4:**
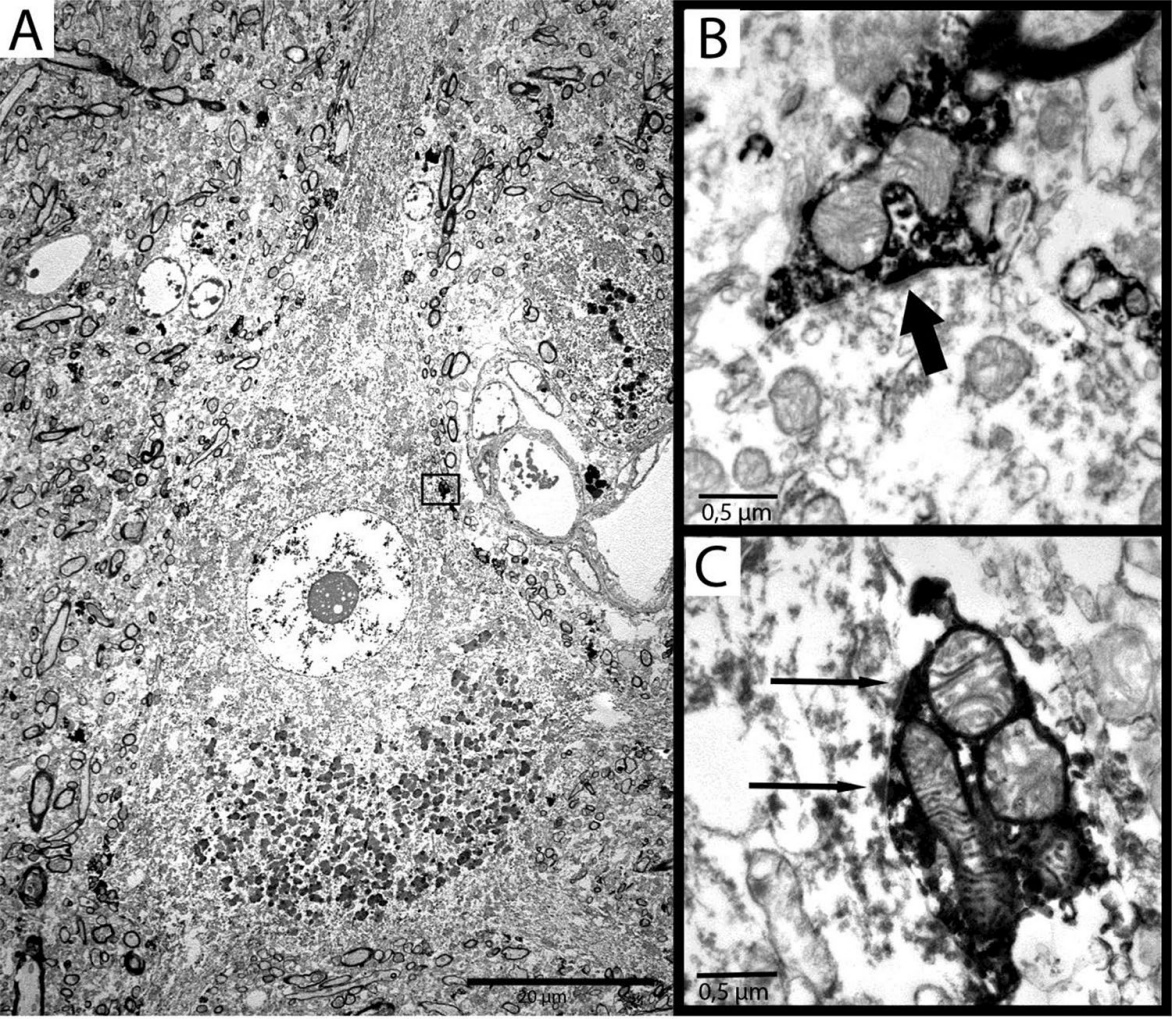
**a** Electron micrograph of a Betz cell and PV-immunopositive perisomatic terminals. **b** The vast majority of PV-immunopositive perisomatic terminals establish symmetric synapses on the somata of Betz cells. Thick arrow shows the synaptic contact. The boxed area in **a** shows a PV-immunopositive perisomatic terminal with asymmetric-like synaptic morphology as it can be seen at high magnification in **c** (thin arrows show the synapses)

Qualitative electron microscopic examinations showed numerous PV-immunopositive terminals around the Betz cells. Most of them showed the morphological features of classical symmetric, presumably inhibitory synapses (Fig. 4) (Gray 1959; Megías et al. 2001). However, some of them displayed asymmetric-like synaptic profile (Fig. 4) on the somata of Betz cells.

**Fig. 5:**
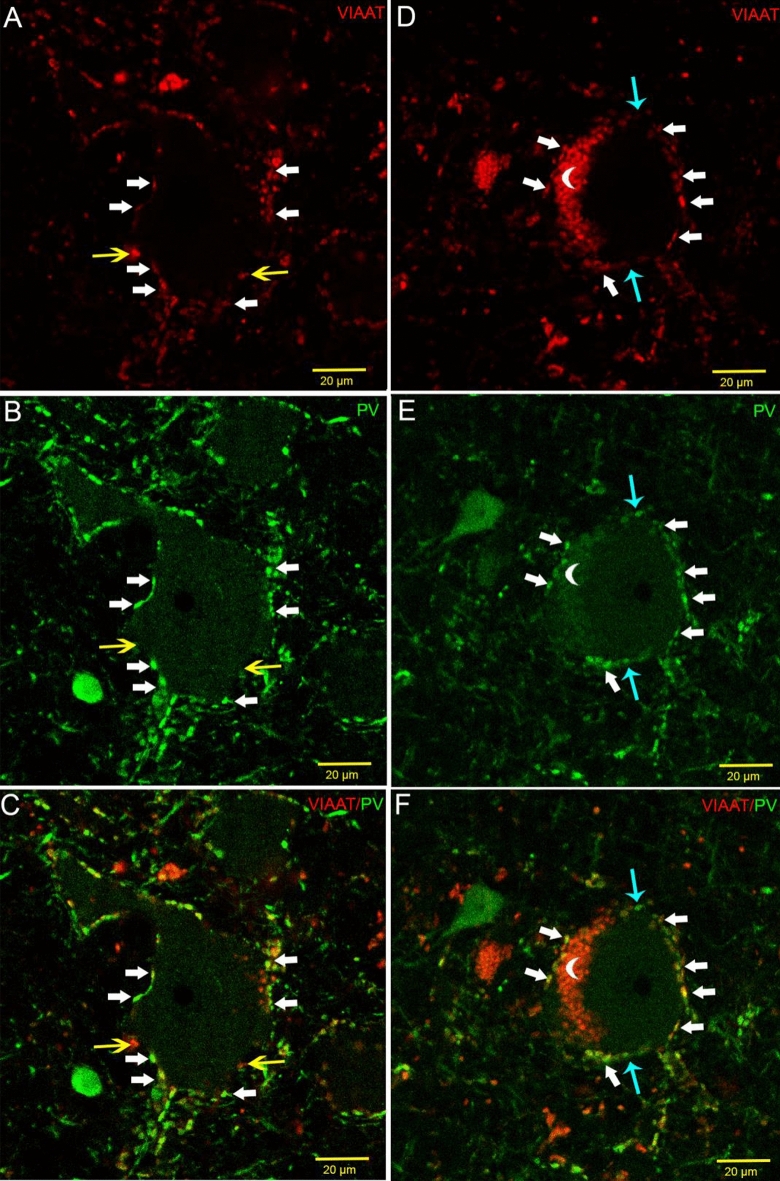
Two giant pyramidal neurons with VIAAT (red) and PV (green) immunofluorescent double labelling. White arrows indicate double-positive terminals, yellow arrows are showing single VIAAT-labelled varicosities, turquoise arrows are pointed to single PV-labelled terminals. According to our analyses, 5.71% of the VIAAT perisomatic terminals are not immunopositive for PV and 4.44% of PV-immunostained terminals are lacking VIAAT immunopositivity. Crescent moon symbols indicate autofluorescent lipofuscin granules

Further analysing the inhibitory nature of perisomatic input to Betz cells, double-fluorescent immunostaining was processed to visualise the VIAAT (vesicular GABA transporter, present in GABAergic terminals) and PV double-immunostained terminals. They co-localised in ~ 90% of the cases (283 terminals out of 315—89.84% in two subjects). The remaining 10% of the terminals were single PV, or VIAAT positive (14—4.44% and 18—5.71%, respectively) (Fig. 5).

**Fig. 6:**
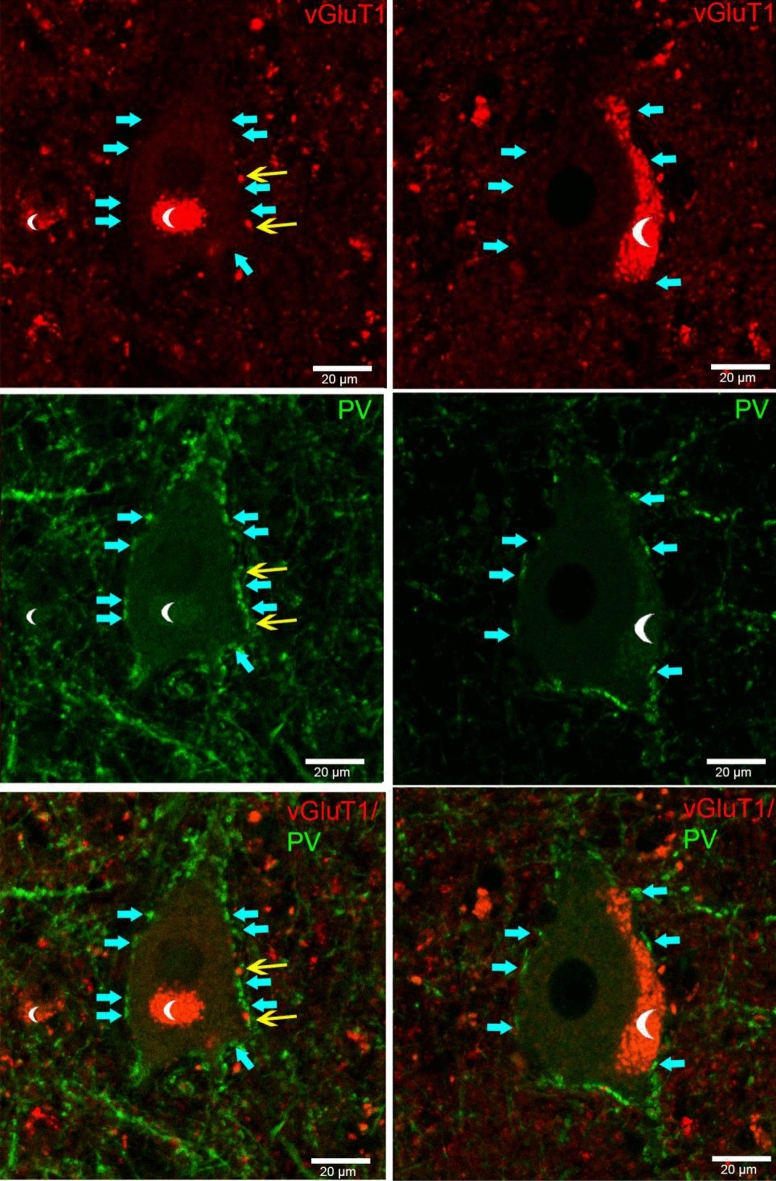
Confocal immunofluorescent images of giant pyramidal neurons with vGluT1 (red) and PV immunostainings (green). There is no co-localisation between PV (turquoise arrows), and vGluT1-immunopositive (yellow arrows) perisomatic terminals. Low numbers of vGluT1-immunopositive terminals are present around the somata of Betz cells, but not on other pyramidal cells. Crescent moon symbols indicate autofluorescent lipofuscin granules

Our question was whether these special parvalbumin-immunopositive asymmetric-like terminals—called “PSD with intermediate thickness” in the substantia nigra (Roberts et al. 2020)—co-express vGluT1 (vesicular glutamate transporter type 1, present in cortical input terminals) or vGluT2 (vesicular glutamate transporter type 2, present in subcortical input terminals). VGluT2-immunopositive terminals were visualised by DAB-Ni reaction, to observe the varicosities and examine them in the electron microscope. Double-immunofluorescent staining with vGluT1 and PV was used to examine cortical glutamatergic input to Betz cells (Fig. 6).

**Fig. 7:**
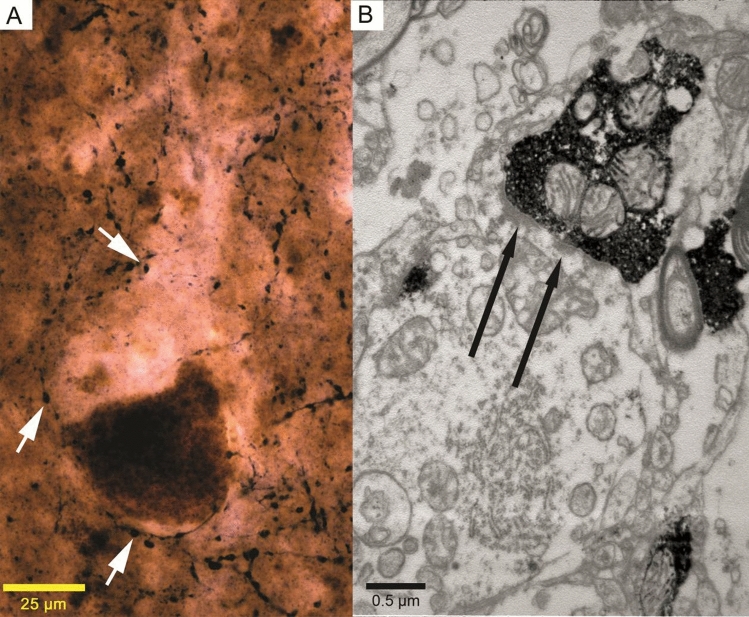
Examination of vGluT2-immunopositive innervation of Betz cells. **a** High-magnification light micrograph of a giant motoneuron surrounded by vGluT2-immunolabeled terminals (white arrows) (40 × objective, stacked picture). **b** High-magnification electron micrograph of a vGluT2-immunolabelled terminal contacting a proximal dendrite. Black arrows show the asymmetric synaptic contacts

VGluT1 and PV were co-localised in less than 2% of the cases (5 terminals out of 310—1.61% in two subjects). Perisomatic contacts of single vGluT1-immunopositive varicosities could be detected in low numbers as well (6 out of 310—1.94%) (Fig. 6). Electron microscopic analysis of vGluT2-immunolabelled terminals revealed that part of the boutons terminated around cell bodies of the Betz cells; however, their vast majority covered the proximal dendrites (Fig. 7).

## Discussion

### Technical considerations

The strength of our results lays in the fact that we were able to use well-preserved human post-mortem samples. The fixation method and the relatively short PMI (3 h 40′ ± 52′) made fine structural analyses possible. However, the relatively small sample size limits the scope of our findings, and we should be cautious to generalise them. Another limitation is that we investigated perisomatic terminals, but inputs to proximal or distal dendrites and the axon initial segment have also important functional roles (Freund and Buzsáki 1996).

### Density and soma size of Betz cells

In concordance with the literature (Tsang et al. 2000), we found that all giant pyramidal cells express SMI32 (Fig. 1). Several studies have carried out stereological investigations on the soma size and density of Betz cells in human subjects and primate (Jacobs et al. 2018; Rivara et al. 2003; Meyer 1987; Lassek 1940; Tigges et al. 1990). The novelty of our investigation is that we used well-preserved samples with 2–4 h of PMI, restricted to the same location inside the primary motor cortex. Therefore, the samples showed homogeneous origin and less post-mortem damage. Nevertheless, we found a considerable diversity among subjects, both in soma size and density.

A previous study (Scheibel et al. 1977) found swelling of the Betz cell bodies besides other changes, like loss of dendrites and dendritic spines during ageing. However, the subjects they investigated were considerably older (between 74 and 102 years). Furthermore, they used materials with 12 h PMI, and post-mortem degradation could cause similar changes in brain tissue (Sele et al., 2019). Therefore, we can assume that the changes they described occur later in life or are the results of poorer preservation. Furthermore, the size of Betz cells in rhesus monkeys seems to be decreasing with age (Tigges et al. 1990).

It is important to emphasise that the subjects investigated in our study differ not only in age, but also in co-morbidities and most likely in lifestyle, too. The amount and quality of physical exercise could significantly affect the preservation and function of the cells contributing to movements as it was studied in an animal model of Parkinson’s disease (Petzinger et al. 2013) or post-stroke patients (Pin-Barre and Laurin 2015). Exercise exerts its beneficial effect on motor function through the BDNF/TrkB pathway (Ploughman et al. 2009). Earlier it was shown in macaque monkeys that Betz cells intensively express TrkB receptors (Ohira et al. 2005) on apical, basal dendrites and cell bodies. Therefore, among others, motor activity can directly affect the number and distribution of giant pyramidal neurons.

### Parvalbumin expression in Betz cells

Studies in non-human primate species (Preuss and Kaas 1996; Ichinohe et al. 2004) showed that Betz cells may contain the calcium-binding protein parvalbumin (PV), but we found no studies investigating the same phenomenon in human samples. Furthermore, these studies report that not all giant pyramidal cells are PV immunopositive, but they did not investigate what proportion of Betz cells expresses PV.

According to our study, most of the Betz cells show PV immunolabelling. It is important to mention that PV immunostaining could fade away after calcium-ion overload as described earlier in epileptic samples (Maglóczky and Freund 1995; Scotti et al. 1997; Wittner et al. 2001). Another important aspect is that PV expression is not so intensive in Betz cells as in PV-immunolabelled interneurons (Ichinohe et al., 2004), which may mean that the signal could be below the detection limit.

PV content of giant pyramidal cells and its lower concentration compared to interneurons could have several functional implications. PV is an effective calcium buffer, potently decreasing the intracellular calcium level while it does not influence the initial ion increase (Schwaller 2010); therefore, relaxation of ion levels is more rapid (Arif 2009). This feature allows PV-containing cells to generate frequent and thin spikes with short duration (Armstrong and Soltesz 2012). As described in macaque monkeys (Vigneswaran et al. 2011), primary motor cortex pyramidal neurons are present this feature. PV content may be necessary for the faster operation compared to other pyramidal cells.

Lower intensity of immunolabelling potentially refers to lower concentration of PV compared to interneurons, which might result in lower calcium-buffering capacity. Further research is needed to clarify functional consequences of this finding, existing data from macaque monkeys (Vigneswaran et al. 2011) show that spike duration is very similar in fast-spiking interneurons and pyramidal tract neurons of the primary motor cortex. Studies of PV knock-out animals show facilitated oscillations of interneurons and Purkinje cells lacking the protein (Schwaller, 2010). Betz cells might be more sensitive for high-frequency firing compared to interneurons.

Interestingly, despite the intersubject variability, we found similar PV-expression profiles in the two hemispheres of the same subjects (Fig. 3). Different PMI and perfusion quality might explain the variation between subjects, but further investigations are needed to explain the difference.

### Investigation of perisomatic synapses

Perisomatic inhibitory terminals are effective tools to control the output of the cells compared to the dendritic inhibition (Freund and Buzsáki 1996; Wittner and Maglóczky 2017). Therefore, in the case of Betz cells, these terminals should have an important role in the execution of fine and precise movements. Accordingly, earlier studies showed that these cells receive dense perisomatic innervation in monkey and human (Sasaki and Iwata 1999; Tigges et al. 1992; Sloper et al. 1978; Marin-Padilla 1972; Gatter et al. 1978).

Two immunohistochemically distinct types of perisomatic terminals are the most frequent contacts of the soma. They originate from cholecystokinin (CCK) or from PV-immunopositive basket cells (Armstrong and Soltesz 2012). Both types have an inhibitory function, use GABA as their neurotransmitter, and give morphologically symmetric, type 2 synapses (Gray 1959).

According to our results, the vast majority of VIAAT-immunolabelled inhibitory terminals around Betz cells are PV immunopositive (89.94%). Therefore, we can assume that PV-immunopositive basket cells have the most important role in controlling the output of Betz cells. In the electron microscope, we found a small number of PV-containing terminals with asymmetric-like or intermediary (Roberts et al. 2020) morphology, which was partially fit the classic synaptic concept of Gray’s (Klemann and Roubos 2011). Previous electron microscopic ultrastructural analyses did not report such phenomenon (Tigges et al. 1992; Gatter et al. 1978) in primate or humans. However, Marin-Padilla (Marin-Padilla 1972) assumed that an ascending thalamic pathway could innervate giant pyramidal neurons. Interestingly, the VL projects to the primary motor cortex (Marlinski et al. 2012), and VL projection cells express PV in humans (Munkle et al. 2000). Excitatory terminals originating from the thalamus contain vGluT2 (Bopp et al. 2017); therefore, it is possible to visualise this subcortical pathway putatively contacting Betz cell somata by vGluT2 immunostaining. In the electron microscope, we were able to observe vGluT2-immunolabelled terminals with asymmetric synaptic contacts (Fig. 7). Perisomatic excitatory input can increase excitability, as described in pathological circumstances (Takahashi et al. 2016). Another excitatory source of perisomatic input can be the 2% proportion of vGluT1-positive terminals found in confocal fluorescent examination on Betz cells’ somata; however, their number may be too low to cause significant effects. In addition, double immunopositivity of 2% of the terminals may be around the detection threshold (Fig. 6).

Despite the low number of subjects, we found moderate heterogeneity in the left–right number, distribution and PV content of giant pyramidal neurons. The low PMI and perfusion-fixation revealed the fine ultrastructural details of the perisomatic innervation of Betz cells. The density of perisomatic innervation of giant motoneurons is extremely high compared to other pyramidal cells. In addition, this somatic input is dominantly PV immunopositive, originating from presumably fast-spiking basket cells (Fig. 5). Our investigation showed further anatomical evidence that the features of Betz cells (e.g. PV content, dense perisomatic inputs, and presumably direct thalamic input) may facilitate a faster functionality, which allows fine motor movements and their rapid correction.
